# Pediatric intensive care unit admissions network—rationale, framework and method of operation of a nationwide collaborative pediatric intensive care research network in Germany

**DOI:** 10.3389/fped.2023.1254935

**Published:** 2024-01-10

**Authors:** Nora Bruns, Christian Dohna-Schwake, Martin Olivieri, Michael S. Urschitz, Susanne Blomenkamp, Clara Frosch, Victoria Lieftüchter, Markos K. Tomidis Chatzimanouil, Florian Hoffmann, Sebastian Brenner

**Affiliations:** ^1^ Department of Pediatrics I, Neonatology, Pediatric Intensive Care Medicine, and Pediatric Neurology, University Hospital Essen, University of Duisburg-Essen, Essen, Germany; ^2^ TNBS, Centre for Translational Neuro- and Behavioural Sciences, University Hospital Essen, University of Duisburg-Essen, Essen, Germany; ^3^ Pediatric Intensive Care Unit, Dr. von Hauner Childreńs Hospital, LMU Munich, Munich, Germany; ^4^ Division of Pediatric Epidemiology, Institute of Medical Biostatistics, Epidemiology, and Informatics, University Medical Centre of the Johannes Gutenberg-University, Mainz, Germany; ^5^ Pediatric Intensive Care Medicine, Department of Pediatrics, University Clinic Carl Gustav Carus, TU Dresden, Dresden, Germany

**Keywords:** pediatric critical illness, pediatric intensive care unit, surveillance, epidemiology, PICU outcome, network, quality control, admission

## Abstract

The Pediatric Intensive Care Unit Admissions (PIA) network aims to establish a nationwide database in Germany to gather epidemiological, clinical, and outcome data on pediatric critical illness. The heterogeneity of pediatric patients in intensive care units (PICU) poses challenges in obtaining sufficient case numbers for reliable research. Multicentered approaches, such as patient registries, have proven effective in collecting large-scale data. However, Germany lacks a systematic registration system for pediatric intensive care admissions, hindering epidemiological and outcome assessments. The PIA network intends to address these gaps and provide a framework for clinical and epidemiological research in pediatric intensive care. The network will interconnect PICUs across Germany and collect structured data on diagnoses, treatment, clinical course, and short-term outcomes. It aims to identify areas for improvement in care, enable disease surveillance, and potentially serve as a quality control tool. The PIA network builds upon the existing infrastructure of the German Pediatric Surveillance Unit ESPED and utilizes digitalized data collection techniques. Participating units will complete surveys on their organizational structure and equipment. The study population includes patients aged ≥28 days admitted to participating PICUs, with a more detailed survey for cases meeting specific criteria. Data will be collected by local PIA investigators, anonymized, and entered into a central database. The data protection protocol complies with regulations and ensures patient privacy. Quarterly data checks and customized quality reports will be conducted to monitor data completeness and plausibility. The network will evaluate its performance, data collection feasibility, and data quality. Eligible investigators can submit proposals for data analyses, which will be reviewed and analyzed by trained statisticians or epidemiologists. The PIA network aims to improve pediatric intensive care medicine in Germany by providing a comprehensive understanding of critical illness, benchmarking treatment quality, and enabling disease surveillance.

## 1 Background and rationale

Childhood critical illness can entail life-long sequelae, necessitating optimal treatment based on high-level evidence. However, clinical research in pediatric intensive care suffers from a heterogenous patient population that brings along difficulties to achieve sufficient case numbers for reliable results (1). Even though pediatric critical illness itself is not a rare disease, many underlying conditions or their combination that cause admission to a pediatric intensive care unit are rare. This frequently requires treatment decisions based on expert opinion rather than evidence, despite international efforts to improve the exploitation of data sources that are available for pediatric intensive care research and foster randomized controlled trials (2–5).

To overcome this obstacle, multicentered approaches are indispensable. In this context, patient registries have proven to be a powerful instrument for collecting large scale data tailored to the particularities of a specific patient population. A few examples of successful registries that include critically ill children are the British PICAnet, Australian/New Zealandian ANZPIC registry, the US American Virtual Pediatrics System, and the NEAR4KIDS data base. In Germany, subgroups of critically ill children are reported to the German TraumaRegister DGU®, German Resuscitation registry and the German Neonatal Network, and the German Burn Registry. Results retrieved from these registries have advanced the field and some have permanently impacted patient care (6–14).

In Germany, intensive care research is clearly underdeveloped in both adult and pediatric care (15). No systematic registration of pediatric intensive care admissions exists, complicating the retrieval of information on the epidemiology, course, and outcomes of pediatric critical illness in Germany. Consistent with the structural underdevelopment of intensive care research, the DIVI (German Interdisciplinary Association of Intensive Care and Emergency Medicine) calls for the implementation of intensive care registries to improve clinical research in this field (16).

Besides the structural requisites for high level clinical research, quality control and disease surveillance structures are insufficient in the field of pediatric intensive care in Germany. At present, it is impossible to assess the quality of care and adherence to treatment guidelines. Further, the pandemic and recent infectious waves of common viruses have revealed that existing disease surveillance structures to early detect rapid changes in disease-specific incidence rates are not suitable to provide timely information to allow rapid response, e.g., by reallocation of resources to manage infectious waves. These shortcomings make it impossible to guarantee the delivery of optimum care for each critically ill child in Germany.

The aim of the Pediatric Intensive Care Unit Admissions (PIA) network is to provide a nationwide database on the epidemiology, course, and short-term outcomes of pediatric critical illness. This protocol describes the framework and method of operation of the proposed PIA network. It is designed as a nationwide observational research network to provide clinical and epidemiological information along with potential indicators of treatment quality and guideline adherence in Germany. Functioning as an open collaborative research network, it grants all contributors the right to conduct research with the collected data. The overarching goal of this project is to create a permanent research network that enables large-scale high-quality clinical and epidemiological research in the field of pediatric intensive care and may possibly serve as the basis for quality control measures in German pediatric intensive care units in the future.

## 2 Methods and analysis

### 2.1 Overarching goals and specific aims

The PIA network was initiated to interconnect pediatric intensive care units (PICUs) in Germany and form a research infrastructure that continuously captures all admissions to these units in a structured manner. It aims to improve the quality of pediatric intensive care in Germany by providing a comprehensive overview of the medical care provided in these units, to identify areas for improvement, to optimize care for pediatric patients, and to enable disease surveillance in pediatric intensive care.

#### 2.1.1 Primary aim

The primary aim of the PIA network is to collect timely nationwide data on diagnoses, treatment, clinical course and short-term outcomes of pediatric critical illness to answer relevant clinical and epidemiological research questions.

#### 2.1.2 Secondary (long-term) aims

After the implementation of quality indicators for pediatric intensive care units, the network may serve as a tool to measure and control treatment quality in PICUs. Suitable indicators are currently being developed by the Association of the Scientific Medical Societies in Germany (AWMF) in collaboration with the German Neonatal and Pediatric Intensive Care Society (Gesellschaft für Neonatologie und Pädiatrische Intensivmedizin, GNPI) and the German Interdisciplinary Association of Intensive Care and Emergency Medicine (Deutsche Interdisziplinäre Vereinigung für Intensiv- und Notfallmedizin, DIVI).

### 2.2 Network, IT-infrastructure, and participation of PICUs

PIA builds on the existing network of children's hospitals, IT-infrastructure, data collection techniques, and regulatory policies of the German Pediatric Surveillance Unit ESPED (www.unimedizin-mainz.de/esped). Established in 1992 to support research activities in the field of rare diseases in the general pediatric population, ESPED is the official disease surveillance and research unit of the German Society of Pediatrics and Adolescent Medicine (DGKJ). ESPED provides the infrastructure to conduct nationwide surveillance studies including almost all children's hospitals in Germany and has advanced the field of pediatrics in various subspecialties by providing otherwise unobtainable data on rare pediatric diseases (17–22).

PIA is coordinated by a steering committee consisting of six individuals from four medical faculties (Essen, Dresden, Mainz, Munich). A local representative of each participating PICU (PIA investigator) ensures data entry and serves as contact person for the network. All German PICUs will be invited to become part of the network, with stepwise PICU enrollment for data entry.

### 2.3 Structural survey of participating PICUs

Upon entrance to the network and annually, a survey on the organizational structure, personnel and equipment of each PICU must be completed by local PIA investigators. The survey is based on the defining characteristics and requirements for PICU levels which are currently being developed by the Association of the Scientific Medical Societies in Germany (AWMF) and will then be made publicly available.

### 2.4 Study population

**Figure 1:**
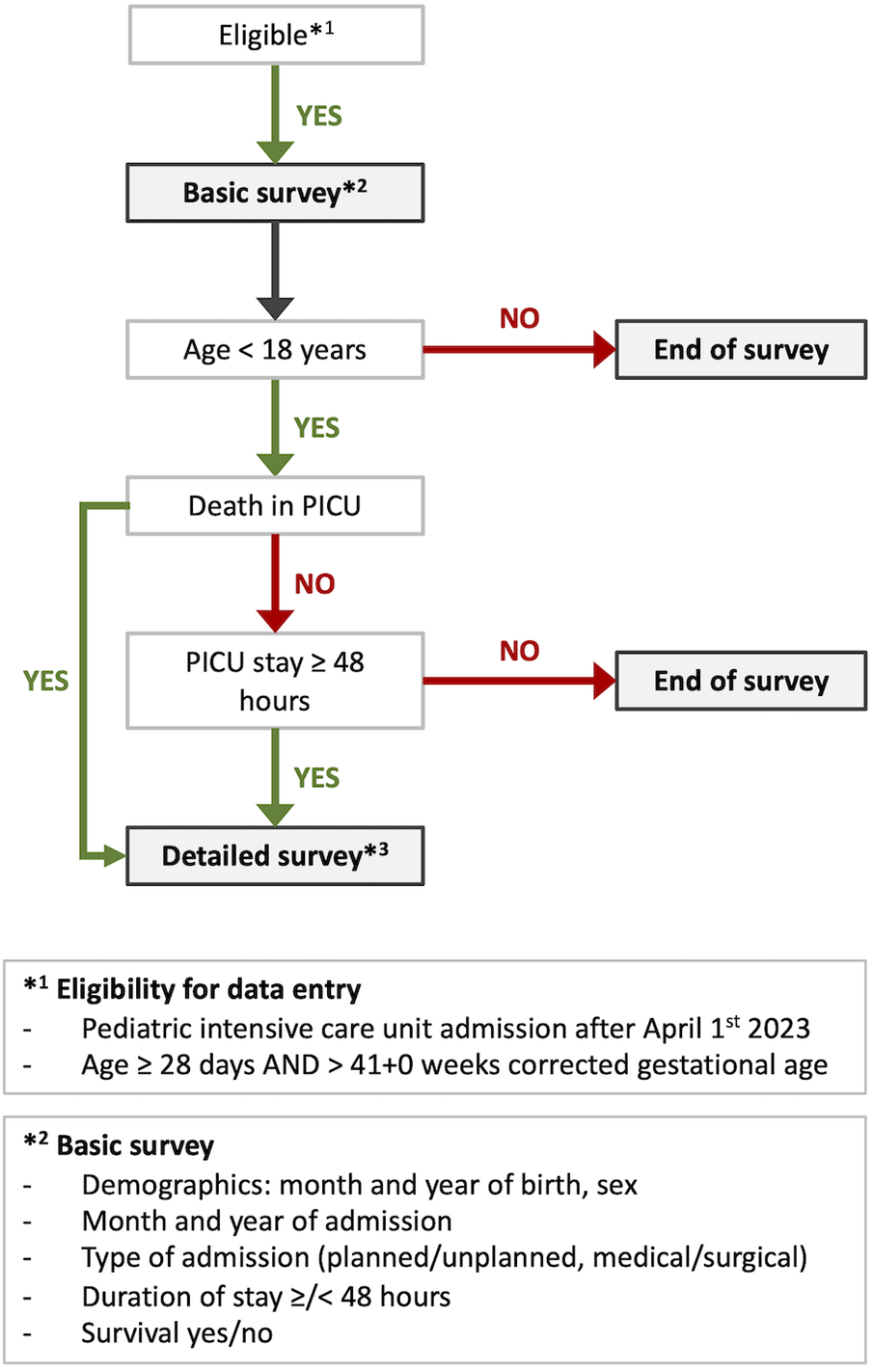
Flow chart of survey allocation for eligible patients to the PIA surveys. PICU, pediatric intensive care unit, *The latest version of the detailed survey is available at https://data.mendeley.com/datasets/nwh3krvz97/1, doi: 10.17632/nwh3krvz97.1.

All patients ≥28 days and >41 + 0 weeks corrected gestational age admitted to a participating PICU are eligible for a basic survey consisting of six items. If criteria for a detailed survey are not fulfilled, data entry is closed after completion of the basic survey (Figure 1). For cases that fulfill the criteria, a more detailed survey will be performed. Criteria for the detailed survey include age <18 years, duration of PICU stay ≥48 h or death within the first two days after PICU admission (Figure 1). Patients discharged from the PICU and readmitted during the same hospital stay are considered new cases. Unplanned PICU readmission of a patient within 24 h is assessed by the local investigators and entered as yes/no into a dedicated variable.

### 2.5 Data collection and data protection

Upon PICU discharge, local PIA investigators enter fully anonymized patient data (i.e., without name, detailed date of birth, home address or other identifiers) via eCRFs into a central database stored on a server at the Institute of Medical Biostatistics, Epidemiology, und Informatics (IMBEI) of the University Medical Centre Mainz (Germany), which serves as data custodian (23).

Patient data are anonymized in a way that reported cases cannot be re-identified, neither by IMBEI personnel nor by scientists analyzing the data. Since only anonymized data from routine clinical care are collected, no informed consent is needed.

The study and data protection protocol were approved by the Ethics Committee at the State Medical Association of Rhineland-Palatinate (study ID: 2022-16893) and the State Representative for Data Protection in Rhineland-Palatinate (study ID: 8223-0001#2023/0002-0104 LfDI). During the course of the project, the entry of the basic survey data will be shifted toward the timepoint of admission in order to comply with demands of the surveillance purpose of the network.

### 2.6 Items

Demographic and clinical variables were drafted and refined by the authors, who are experts in the fields of pediatric intensive care and pediatric epidemiology, until consensus was achieved. After testing the practicability and feasibility of the proposed survey on real cases at the University children's hospitals of Dresden, Essen, and Dr. von Haunersches Kinderspital Munich, three more rounds of refinement including literature search and expert discussions were conducted.

The definitions of variables (data dictionary) are deposited in English and German language at the homepage of the PIA network and Mendeley data (doi: 10.17632/nwh3krvz97.1). Updates of the variable list will be deposited for maximum transparency and to provide common data elements for use in pediatric intensive care research.

### 2.7 Planned refinement of variables during ongoing data collection

After the first year of data collection, data will be assessed for completeness and plausibility and local investigators of the participating PICUs will be queried for potential improvement of variables. Selected variables will then be revised as appropriate, and local PIA investigators queried regularly to continuously improve the registry.

### 2.8 Data monitoring and quality assurance

Collected data are checked for completeness and plausibility on a quarterly basis (i.e., 4-times per year in April, July, October and January for the respective past quarter). Customized center-specific data quality reports are generated and sent to the PIA steering committee and local PIA investigators. Lack of completeness or plausibility (e.g., >2% of missing/implausible values) are discussed and possible solutions elaborated (e.g., improvement of survey items or the e-CRFs).

### 2.9 Steps of the evaluation

According to the early stage of the development of the PIA network (including structures and processes), proof-of-concept aspects will be evaluated in a first step. This includes performance of the network, feasibility of data collection and quality of collected data. After this proof, data will be evaluated concerning their potential in the fields of clinical research, quality control and disease surveillance (see below).

### 2.10 Assessment of survey methodology

The quality of the survey methods is investigated on an annual basis (i.e., for the preceding calendar year). Quality indicators comprise (i) the nationwide coverage of the PIA network (based on eligible and participating centers), (ii) the completeness of reported cases (by comparing PICU admissions with the number of cases captured by the central database), (iii) the completeness of values per case and possible reasons for incompleteness and (iv) the amount of and reasons for implausible values.

For the latter two, multi-level regression analysis will be used with binary indicators for missingness and implausibility as dependent variables and PICU-related context factors and individual demographic and clinical characteristics of cases as independent variables.

### 2.11 Data sharing, accessibility and publication policy

Each participating center has the right to analyze entered data from the own center at any time without restrictions.

Proposals for data analyses of the complete data set can be submitted by eligible investigators specified in a publication guideline. Briefly, all local PIA investigators and members of the steering committee are entitled to submit a proposal. Each proposal will be evaluated for methodological feasibility and scientific relevance by an internal review board. After endorsement, the data will be analyzed by the designated statistician/epidemiologist in collaboration with the initiators of the study. A manuscript draft must be submitted to the internal review board for approval within one year after the initial endorsement. After approval by the internal review board, the manuscript can be submitted to a journal for publication.

### 2.12 Data analysis

Data analysis is only carried out by trained statisticians or epidemiologists using the appropriate methods to answer the respective research question. In general, effect estimation is preferred over statistical hypothesis testing whenever appropriate. The original dataset will not be passed on to investigators or made publicly available.

### 2.13 Annual reports

Reports of the network will be published annually. An anonymized ranking with relevant PICU outcomes will be provided to each PICU to promote benchmarking and identify fields for potential improvement.

### 2.14 Affiliation with medical societies

The PIA network is officially endorsed by the GNPI and the Pediatric section of the DIVI.

### 2.15 Funding

The conceptualization of the network was kindly supported by the Stiftung BINZ (Ulm, Germany). For the nationwide roll-out and permanent consolidation of the registry, additional funding will be sought.

## 3 Discussion

The presented PIA network aims to improve pediatric intensive care medicine in Germany on three levels: For the first time, it will create the possibility to conduct nationwide observational studies on critically ill children, measure and compare treatment quality between pediatric intensive care units, and improve disease surveillance. The initiators' network in the field of pediatric intensive care, experience with multicentered and nationwide surveillance studies, the existing ESPED infrastructure that requires only adaptation instead of a new setup, and broad acceptance of ESPED among pediatricians are prerequisites that promote a successful implementation of the network into the pediatric intensive care landscape in Germany. All criteria for successful PICU registries published by Wetzel (24) are fulfilled.

Worldwide, networks and registries are acknowledged as powerful instruments to conduct research in specific patient populations. In pediatric medicine, several registries that include critically ill children have been established to closer monitor subpopulations, e.g., neonates or injured children: The German Neonatal Network (GNN) has strongly influenced neonatal practice by continuously providing evidence on benefits of treatment interventions and unveiling previously unknown associations between risk factors and diseases (10, 25–27). Likewise, the TraumaRegister DGU® allows in-depth analyses of severely injured children on all aspects of pre-hospital care, shock room management and the subsequent intensive care unit stay (28–31). The impact of the obtained results has reached far beyond scientific purposes but lead to adjustments of emergency care structures and processes. Deep insights into the airway management of critically ill children have been obtained from the National Emergency Airway Registry for Children (NEAR4KIDS) database located in the United States (13, 32, 33). Nationwide projects on critically ill children in the actual sense of PICU registries are the Australian and New Zealand Paediatric Intensive Care Registry (ANZPICR) (33–35) and the British PICAnet, which frequently outputs high impact research (9, 11, 36–38).

With these influential networks and registries as role models, the PIA network aims to contribute a building block to advance pediatric intensive care research and ultimately improve the treatment of critically ill children. Nationwide data on all aspects of pediatric critical illness in Germany will be collected and made available for research purposes. This will unveil different treatment strategies between centers and enable comparisons regarding short-term outcomes and complications. With Germany being one of the European countries with the largest population, findings from the PIA network may bring along important insights on an international level and advance population-level pediatric intensive care research. Considering the recent call of the DIVI to establish intensive care registries for research purposes, the PIA network excels this claim with its goals of improving inter-PICU networking, disease surveillance, and quality control.

In the future, the different levels of PICUs and their contribution to pediatric intensive care provision in Germany can be characterized with the help of PIA for the first time. Annual reports will benchmark PICUs to identify their own strengths and weaknesses to optimize patient care accordingly. The assessment of patient outcomes makes it possible to measure the quality of care and provides the basis for potential PICU certifications in the future. As a beneficial side effect, the fact that outcomes are measured may increase awareness for the need to follow-up PICU patients. Even though not directly recorded, this may also bring into consciousness the recently described post-PICU syndrome (39, 40), hopefully fostering the implementation of structured PICU aftercare programs.

Despite thorough planning, the PIA network has some limitations that could not be avoided at the time of conceptualization. The largest limitation is the need for retrospective manual data entry, which further burdens the already-limited human resources in German hospitals. For that reason, the patient population that is monitored in detail is limited to severely ill children. Less severely ill children with PICU stays shorter than 48 h will only be registered with a short survey. Also due to limited resources, follow-up is only short-term, potentially missing out on important long-term sequelae among PICU survivors. In order to not interfere with neonatal registries (German Neonatal Network, Hypothermie Registry), only non-neonatal cases are eligible for the PIA network. This may cause certain subgroups, such as infants with congenital malformations, to remain uncaptured by either registry and require refinement in the future. Because participation in the network is voluntary, a comprehensive surveillance of all German PICUs and all admitted patients will likely remain unachievable. The local PIA investigators are responsible for data entry and no monitoring is available at the timepoint of the network's implementation to ensure the completeness and quality of entered data - close interaction with the participating centers will be maintained in order to encourage active participation in the network. Further, no government- or institutional funding is available at the timepoint of implementation, putting the long-term continuation at risk.

To overcome these limitations, the PIA network will require constant refinement and advancement of methods. For example, as much data as possible should be automatically transferred to minimize documentation efforts of PICU staff. This requires the consequent pursuit of digitalization which should include the data integration centers located at German university hospitals. With ongoing digitalization of hospital documentation, automated data export may reduce the burden of manual data entry, for example by designing PIA-compatible digital admission forms, extracting routine electronical patient documentation or hospital billing information. These processes will also be fundamental cornerstones to achieve and maintain high up-to-dateness of the registry. To enable prompt reactions, e.g., to infectious waves, real-time or near real-time information is indispensable. The authors’ vision is to further develop the registry towards a real-time monitoring tool that represents the current state of pediatric intensive care utilization along with important real-time information of public interest in a dashboard on the homepage of the PIA network.

In summary, the PIA network is a well-planned nationwide pediatric intensive care network and registry that envisions to improve the care for critically ill children in Germany in terms of improved research opportunities, quality measurement, and enhanced surveillance of PICU resource utilization. The organizational embedding into the long-established and acknowledged structures of the national surveillance unit ESPED, which belongs to the German Society of Pediatrics and Adolescent Medicine, and the ideational support of the two major German medical societies involved in the care of critically ill children make a successful implementation likely. However, the future success and long-term continuation of the network will depend on its ability to motivate PICU practitioners to engage in the network and to realize technological advancements to facilitate data acquisition.

## Ethics statement

According to local legislation, the retrospective collection of anonymized data from clinical routine care does not require informed consent or the approval of an institutional review board. Despite this, a waiver was obtained from a local Ethics Committee (study ID: 2022-16893).
